# Nightmares in Children with Foetal Alcohol Spectrum Disorders, Autism Spectrum Disorders, and Their Typically Developing Peers

**DOI:** 10.3390/clockssleep3030033

**Published:** 2021-09-16

**Authors:** Rabya Mughal, Siu Sing Wong, Dagmara Dimitriou, Elizabeth Halstead

**Affiliations:** Sleep Education and Research Laboratory, Department of Psychology and Human Development, University College London Institute of Education, London WC1H 0AA, UK; sam.sswong@yahoo.com (S.S.W.); d.dimitriou@ucl.ac.uk (D.D.); l.halstead@ucl.ac.uk (E.H.)

**Keywords:** sleep, neurodevelopment, nightmares, dreaming, autism, foetal alcohol spectrum disorders

## Abstract

Children with Foetal Alcohol Spectrum Disorders (FASD) and Autism Spectrum Disorders (ASD) experience significantly higher rates of sleep disturbances than their typically developing (TD) peers. Pre-sleep anxiety and waking emotional content is known to affect the content and frequency of nightmares, which can be distressing to children and caregivers. This is the first study to analyse nightmare frequency and content in FASD, and to assess its association with psychometric outcomes. Using online caregiver questionnaires, we assessed reports from 277 caregivers of children with ASD (*n* = 61), FASD (*n* = 112), and TD children (*n* = 104) using the Children’s Sleep Habits Questionnaire (CSHQ), the Child Behaviour Checklist (CBCL), the Spence Children’s Anxiety Scale (SCAS), and the Behaviour Rating Inventory for Executive Functioning (BRIEF). Within the ASD group, 40.3% of caregivers reported their children had nightmares. Within the FASD group, 73.62% of caregivers reported their children had nightmares, and within the TD group, 21.36% of caregivers reported their children had nightmares. Correlation analysis revealed significant associations between anxiety and nightmares, maladaptive behaviour and nightmares, and executive functioning and nightmares in the TD and FASD groups, but not ASD group. This paper adds to the emerging body of work supporting the need for sleep interventions as part of clinical practice with regard to children with ASD and FASD. As a relatively niche but important area of study, this warrants much needed further research.

## 1. Introduction

During rapid eye movement (REM) sleep, it is thought that brainstem generators physically activate motor, perceptual, affective, cognitive, and amnestic circuits whose information is then synthesised into what is known as dreaming [1]. The sequential operation of non-REM (NREM) and REM sleep throughout the night is crucial for a fully functioning cognitive system [2,3], and is fundamental to the cognitive and emotional development of the child [4].

Nightmares are defined as disturbing mental experiences that awaken the dreamer and generally occur in REM sleep [5,6,7,8,9], alongside typical negative emotions of terror, fear, and anxiety [9,10,11,12,13]. First nightmare experiences tend to be at around three years old, peaking between six and ten years old [14,15,16]. The nightmare frequency of children is also found to be associated with higher levels of anxiety, as rated by caregivers [17]. Furthermore, nightmare frequency is associated with the mood regulation function of dreaming [18], suggesting nightmares occur as a result of intense pre-sleep negative emotions. Moreover, nightmares may prompt bedtime worries and anxiety and can therefore result in bedtime resistance behavioural problems [17], such as leaving the bedroom, stalling bedtimes, or the need to fall asleep next to the parent [4]. It is also reasonable to note the bidirectional relation between nightmare frequency and child anxiety [18,19]. Indeed, night time fears, anxiety, and recurrent nightmares are commonly presented problems in paediatric sleep, particularly in children with significant trauma [20]. In paediatric populations, negative affect and trauma is connected to both associative and hyperassociative nightmares [21].

Younger typically developing (TD) children tend to have more active imaginations, and are likely to have fantastic-associated nightmares (such as zombies, monsters, witches) whereas older TD children and adolescents are more likely to have realistic and wake-congruous nightmares (such as school, academic performance, friendship problems) [20]. Meanwhile, the age-related changes in children’s dreams appear to parallel emotional and cognitive development, since the older that children become, the more they are able to describe the frequency and length of dreams and provide detailed descriptions [22,23]. Nightmares are associated with intense waking negative affect, socioeconomic criteria (such as family income), and psychometric outcomes, such as frequent temper outbursts, mood disturbance, hyperactivity, as well as school performance [24]. This raises the question of whether nightmares play a role in children for whom there is an atypical cognitive, social, and emotional developmental trajectory. In children with neurodevelopmental conditions, there is a scarcity of data on nightmare content, frequency, and hyperassociation; however, neural stress responses and their overlap with sleep-arousal mechanisms may be a factor in the significant overlap between sleep and psychometric outcomes [25]. Sleep-related neurotransmitters, including GABA, CRH, and sleep-arousal mechanisms including the HPA axis are known to be developmentally compromised in the clinical conditions examined within this paper, and may be part of the complex interaction between sleep and psychometric outcomes in children with Autism Spectrum Disorders (ASD) and Foetal Alcohol Spectrum Disorders (FASD) [26]

ASD is a combination of neurodevelopmental conditions characterised by social and communication difficulties, repetitive behaviour, and sensory sensitivities [27]. Children with ASD have a higher occurrence of sleep disorders, anxiety, and emotional regulation difficulties than their TD peers [28], which is associated with cognition and affect in this population [29]. Insomnia, parasomnias, and disrupted sleep are significantly higher in ASD [30] and significantly associated with hyperactivity, aggression [31], affect [32] behavioural and social adaptive difficulties [33], cognition [34], communication difficulties [35], and academic performance [36]. However, the phenotypic presentation of nightmares in this population is less clear. Adults with ASD are reported to experience fewer nightmares than controls [37], whilst in paediatric ASD populations the incidence of parasomnias is higher [29] but nightmare frequency is variable in comparison to controls [37]. One study with 345 children with ASD, controls and siblings demonstrated that siblings were more likely to experience nightmares than controls or the clinical population [38].

Foetal Alcohol Spectrum Disorder (FASD) is the neurodevelopmental and physical consequence of prenatal alcohol exposure, manifesting in a pattern of executive functioning, social functioning, sensory perception, and learning difficulties. Syndromic physical features may also be present [39]. Children with FASD are significantly more likely to experience trauma, attachment disorders, and anxiety disorders than TD children. This is the result of both pervasive cortical thinning due to prenatal alcohol exposure as well as epigenetic influences as a result of environmental stressors [40]. Around 80% of children with FASD are in the care of adults other than the biological parent, which contributes to attachment, attentional, and other pervasive neurodevelopmental problems [41]. Sleep problems are prevalent in children with FASD and are thought to have a bidirectional association with neuropsychological outcomes, such as sensory sensitivity [42], anxiety [43], and cognition [44]. Caregiver reports reveal that children with FASD present with significantly more sleep problems than TD children [45]. Caregiver report, polysomnography (PSG), and actigraphy studies report that children with FASD have a significantly shorter sleep duration, lower sleep efficiency, more sleep disruption and more sleep disordered breathing, and higher nightmare frequency than TD children [45].

The purpose of this study was to take an exploratory approach to assess the content of nightmares and their associated outcomes. Our aims were to report whether children with the two neurodevelopmental conditions, ASD and FASD, were more likely to experience nightmares, and whether there is a correlational trend between nightmare content and psychometric outcomes of affect, behaviour, or executive control.

## 2. Materials and Methods

### 2.1. Ethical Approval

This study was approved by the UCL Institute of Education Research Ethics Committee (Approval number 16683/001). All caregiver participants gave written informed consent.

### 2.2. Questionnaires

This study used several questionnaires to assess parental reported sleep and daytime functioning, and relevant background information of the child.

Children’s Sleep Habits Questionnaire (CSHQ; [46]): A 33-item caregiver report which screens for common sleep problems in school aged children. It is rated on a 3-point scale (‘rarely’ for an event that occurs 0–1 times per week; ‘sometimes’ for an event that occurs 2–4 times per week; ‘usually’ for an event that occurs between 5–7 times per week). The CSHQ is a widely used assessment tool in paediatric sleep, with high internal validity and Cronbach alpha score of 0.83 [47]. Items are grouped into eight subscales: bedtime resistance, sleep onset delay, sleep duration, sleep anxiety, night waking, parasomnia, sleep disordered breathing, daytime sleepiness. Clinical scores are determined as 41 or above. Nightmare frequency and content were taken from Subscale Item 6: Parasomnias (“Does your child have nightmares? If so, describe the frequency and content”).

Spence Children’s Anxiety Scale (SCAS; [48]): A 39-item caregiver report which screens for clinical anxiety levels in children aged between 6 and 16. It is rated on a 4-point scale (0 = never to 3 = always). The SCAS is a widely used instrument with high internal validity and a Cronbach’s alpha score of 0.92. The SCAS assesses various anxiety symptoms specified in the DSM-V, including subscales for separation anxiety, social phobia, obsessive-compulsive disorder, panic, physical injury fears, and generalised anxiety disorder. Clinical scores are determined as 31 and above [49].

Behaviour Rating Inventory of Executive Function (BRIEF; [50]) An 83-item caregiver report which screens for atypical executive functioning in children aged between 5 and 16 years. It is rated on a 3-point scale (1 = never to 3 = often). The BRIEF is a widely used measurement of executive functioning with a Cronbach alpha score of 0.8–0.98. Eight aspects of executive functioning are assessed. Subscales are working memory, inhibition, shifting, emotional control, and planning and organisation. Clinical scores are determined as 65 and above [50].

Child Behaviour Checklist (CBCL; [51]). A 118-item caregiver report which measures maladaptive behaviours in children aged between 5 and 16. It is rated on a 3-point scale (0 = not true to 2 = very true). The CBCL is widely used in research settings with mental health workers, hospital staff, foster parents, clinicians, and teachers. It has high internal validity, with a 0.94 Cronbach’s alpha score. Subscales are divided into ‘Internalising’ and ‘Externalising’ behaviours, as well as the subsets of: withdrawn, somatic complaints, anxious/depressed, social problems, thought problems, attention problems, delinquency, and aggression. Clinical scores are determined as a total of 64 and above [52].

The Childhood Autism Rating Scale, Parents Version (CARS; [53]). A 15-item caregiver questionnaire that screens for the severity of autistic symptoms, using a seven-point (including midpoint) Likert Scale, ranging from typical to atypical behaviour. Categories are: relating to people, imitation, emotional responsiveness, body use, object use, adaptation to change, visual responses, listening responses, taste, smell, touch responses, fear or nervousness, verbal communication, nonverbal communication, activity levels, intellectual responsiveness, and general observations. The CARS demonstrates moderate to good sensitivity and specificity (81.4% and 78.6%, respectively) and good internal consistency (Cronbach’s alpha = 0.79) however cannot be used in place of a diagnostic assessment. A CARS score of > = 33 indicates possible ASD [54].

Neurobehavioural Screening Tool (NST; [55]). A ten-item binary checklist that screens for possible FASD in children. Questions examine whether children meet the more common neurobehavioural characteristics of FASD, however these are not always accurate or representative of all children with FASD. Categories are: acting young, lying and cheating, lacking guilt after misbehaving, difficulty concentrating, impulsivity, hyperactivity, displays of cruelty, stealing at home, and stealing outside of home. The NST has low sensitivity but high specificity (62% and 100%, respectively) and in the absence of a more accurate measurement tool, is a widely used screening mechanism for FASD in children. Scores above 8, plus confirmed prenatal alcohol exposure indicate that a FASD diagnostic evaluation should be conducted [55].

### 2.3. Participants

Participants were caregivers of children aged between 6 and 15 years old (between the 6th and 16th birthday). This age group was chosen since it represents a large age range from which a cross sectional analysis could be made.

All children had received either a diagnosis of FASD or ASD, and no diagnoses of any physical or mental health conditions in the TD group. Screening tools for FASD and ASD were additionally included in the battery of questionnaires that caregivers completed. A number of children also had secondary diagnoses, which are outlined in Table 1 below.

This study was initially advertised as an online questionnaire assessing sleep and daytime functioning in children with FASD or ASD. Caregivers of TD participants were recruited through three schools in West London, South London, and Milton Keynes. Caregivers of children with FASD were recruited through the UK FASD Network mailing list, while caregivers of children with ASD were recruited through online ASD forums. Caregivers were directed to an online questionnaire, at the end of which there was an option to receive feedback on the child’s score as well as sleep hygiene intervention ideas.

A total of 322 caregivers completed the online questionnaire. Nine responses were excluded as large sections of the questionnaire were not completed. Twenty-four responses were excluded as they did not have a diagnosis of FASD or ASD, and were not TD. A further 12 were excluded as they did not meet the age criteria. One-way between group ANOVAs indicated no age (F(1,235) = 1.06, *p* = 0.43, η_p_^2^ = 0.85) or SES differences (F(1,3) = 1.06, *p* = 0.49, η_p_^2^ = 0.01), but there were significant differences between sex, with significantly more boys than girls (F(1,2) = 6.58, *p* = 0.01, η_p_^2^ = 0.02). The final sample consisted of 277 participants, outlined in Table 1.

### 2.4. Statistical Analysis

We performed quantitative methodology using SPSS Statistics for Windows, Version 23.0 (IBM SPSS Statistics for Windows, Version 23.0. Armonk, NY, USA: IBM Corp). The questionnaire had a 92% response rate with 146 missing values. Where there were missing data, these were imputed in SPSS. One-way ANOVA with Gabriel post-hoc tests were conducted to examine group differences of nightmare frequency, child sleep habits, child anxiety, executive function, and behaviour. Pearson’s correlation coefficient was performed to test if the nightmare frequency was associated with clinical outcome measures. Qualitative content analysis was conducted using NVivo V.12 on novel items, which could not be categorised into the Garfield [56] and Shredl and Pallmer [21] categorisation of ‘being chased’, ‘injury or death’, or ‘sensing scary’.

## 3. Results

### 3.1. Thematic Analysis of Nightmare Themes

Table 2 shows the percentage of children whose nightmare content fell into the categories of ‘being chased’, ‘injury or death’, and ‘sensing scary’, as reported by Garfield [56] and Schredl and Pallmer [21].

Table 3 shows the characters each group experienced within the three themes. Children in the TD group reported being chased by a ‘dog’ whilst children in the clinical groups reported being chased by more fantastic characters such as ‘aliens’, ‘monsters’, or ‘large insects’. Similarly, whilst TD children reported dreaming of the death of ‘people’ and ‘puppies’, the children from the ASD and FASD groups had dreamed of the death of unusual characters like ‘toys’ and ‘aliens’, or significant family members. Children in the FASD group were the only ones to dream of concrete characters (e.g., witches) in the nightmare theme of ‘sensing scary’.

Table 4 presents the results of the thematic analysis based on the nightmare themes of Gakenbach [57] and Revonsuo and Salmivalli [58]. Nightmare content from the FASD group was classified into the theme of fantasy (30%), persecuted/being chased (18%), and robbery/theft/crime (9%), but less for the theme of real life (9%), while the pattern was somewhat similar between the TD and ASD groups. A full list of nightmare content can be found in the Appendix A.

### 3.2. Group Comparison on Nightmare Frequency and All Clinical Outcome Measures

One-way ANOVA with the Gabriel post-hoc test was conducted to examine if nightmare frequency, child sleep habit, child anxiety, behaviour, and executive function varied between the three groups. Under the timeframe from ‘less than monthly (1)’ to ‘nightly (5)’, nightmare frequency was significantly varied among the three groups (F(2, 58) = 7.142, *p* = 0.002, η^2^ = 0.203), with the children from the TD group (M = 2.25, SD = 1.06) scoring significantly lower in nightmare frequency than the ASD group (M = 3.57, SD = 1.09, *p* = 0.009) and the FASD group (M = 3.58, SD = 1.09, *p* = 0.001), while there were no differences of nightmare frequency between the ASD and FASD groups.

As Table 5 shows, the omnibus F test of one-way ANOVA showed the significant group differences on the total CSHQ (F(2, 115) = 6.842, *p* = 0.002, η^2^ = 0.108), total SCAS (F(2, 115) = 6.842, *p* = 0.002, η^2^ = 0.108), total CBCL (F(2, 115) = 19.735, *p* < 0.001, η^2^ = 0.259), and total BRIEF (Welch’s F (2, 40.867) = 22.018, *p* < 0.001). Specifically, the results of the Gabriel post-hoc test indicated that the children in the TD group scored significantly lower than both the ASD and FASD groups, whilst there were no differences found between the ASD and FASD groups. These patterns of findings were also found in subscales of the CSHQ, SCAS, CBCL, BRIEF, but not the obsessive-compulsive scale of the SCAS, or withdrawn, anxious/depressed, social problems, delinquency externalising behaviours, and aggression externalising behaviours within the CBCL.

### 3.3. The Relation between Nightmare Frequency and Clinical Outcome Measures

Pearson’s correlation coefficient with bootstrapping was conducted to examine whether nightmare frequency was associated with any of the clinical outcome measures in each group of children. As Table 6 shows, the nightmare frequency of TD children was positively significantly associated with maladaptive behaviour and executive function, while the nightmare frequency of the children from the FASD group was positively significantly related to child maladaptive behaviour and anxiety. No correlations were found withing the ASD group.

## 4. Discussion

This is the first study to explore the associations between nightmares and psychometric outcomes in children with ASD, FASD, and in TD children. We aimed to explore nightmare frequency and content among the three groups, as well as correlational associations between nightmare frequency, anxiety, maladaptive behaviour, and executive functioning in children with ASD, children with FASD, and TD children. Children in both clinical groups scored significantly higher on anxiety, maladaptive behaviour, and executive functioning measures than their TD peers. Children in both clinical groups experienced significantly more sleep problems than the TD group. Within our sample, children with ASD and FASD had significantly more nightmares than TD children: seventy-four percent of children in the FASD group were reported to have nightmares; 40% of the ASD group were reported to have nightmares, which was in comparison to 21% reported in the TD group. Nine percent of the TD group reported to have weekly nightmares, which is comparable to previous data which reported weekly nightmares in 5.2% of a sample of 6359 TD children [26]. In addition, we report that within our sample nightmare frequency was significantly associated with maladaptive behaviour and anxiety in children with FASD. Nightmare frequency was also significantly associated with maladaptive behaviour and executive functioning difficulties in TD children. These results demonstrate much needed further investigation into this area of research.

### 4.1. Nightmare Content

Children in the TD group were more likely to report nightmares involving insects and dogs. This is in line with previous studies in TD populations, reporting frequent appearances of animals in children’s dreams [22]. Children in the ASD group reported more death, injury, and violence related nightmares than the TD group, but also similar amounts of real life, being chased, fantasy, and separation related nightmares. Children in the ASD group were more likely to report nightmares of themselves being injured, whereas children in the TD and FASD groups were more likely to report nightmares of significant others being injured. In one comparison study [38] between children with ASD, siblings, and a control group (n = 345), it was noted that the experience being raised, i.e., having more than one caregiver and changing caregivers frequently, was positively associated with nightmare frequency. This could perhaps be an explanation for the higher number of appearances of attachment figures within the FASD nightmares, as well as higher frequency of nightmares in the FASD group. Children in the FASD group reported the most robbery, theft and crime related nightmares, persecutory nightmares such as being chased (usually by wolves, monsters, or strangers), separation nightmares, and nightmares involving people, toys or animals with significance being injured, dying, or taken away. According to the Threat Simulation Theory, threatening experiences during wake are rehearsed during sleep, possibly due to dream consciousness working as an evolutionary defence mechanism, in which the brain repeatedly simulates threatening events [58]. Children with FASD are significantly more likely to experience attachment issues as a result of upbringing differences, including the frequent changing of caregivers. This may explain why threatening situations, such as being chased or family members dying were rehearsed by children with FASD. Meanwhile, injury to self was more prominent in children in the ASD group which could perhaps be due to the ‘self-other’ perception within ASD [59].

### 4.2. Nightmare Frequency and Psychometric Associations

In the TD group, nightmare frequency was significantly associated with composite CBCL scores, as well as the subscales of anxious and depressed behaviour, attentional problems, and aggression. Nightmare frequency was significantly associated with composite BRIEF scores, as well as the subscales of working memory, shifting, planning and organising, initiation, and emotional control. In the FASD group, nightmare frequency was significantly correlated with composite CBCL scores, as well as the subscales of withdrawn, somatic, anxious/depressed, and thought problems. Nightmare frequency was also significantly associated with total SCAS as well as the subscale of panic symptoms. No associations were found within the ASD group. One narrative review [37] has indicated the positive relation between nightmare frequency and psychiatric outcomes such as posttraumatic stress, depressive, bipolar, anxiety, and obsessive compulsive disorders, but not in ASD where they report the association between nightmares and psychometric outcomes is less clear. In this narrative review, adults with ASD were reported to have significantly fewer nightmares than controls, and there appeared to be no relationship between ASD and nightmares in children and adolescents [37]. Inconsistent to previously reported data on nightmares in children with ASD, our study reports that children with ASD experienced a significantly higher number of nightmares than their TD peers. The frequency of nightmares has previously been positively associated with ASD symptomology and IQ [31], but not to psychometric outcomes, whilst parasomnias in general have previously been reported to have various associations with psychometric outcomes, some positive, some non-conclusive [29]. This is consistent with our findings. This nonlinear relationship may be due to lower scores on the CBCL, SCAS, and BRIEF in contrast to the ceiling effect noted below.

This lack of association in the ASD group may also be due to the processes involved in emotional regulation and hypersensation. Higher emotional regulation difficulty is associated with higher nightmare frequency in light of the influence of waking intense emotions [60], and the theory of the adaptive emotion regulation function in dreaming [22]. Based upon these findings, emotion regulation difficulty could be the underlying factor for the nightmare frequency in the ASD group, in which difficulties in emotion regulation are well reported [28]. In addition, hypersensitisation in ASD may be a contributory factor. Early adverse experiences during sensitive windows can have a long-lasting effect on nightmare frequency, that is, a trait variable maybe associated with sensory processing sensitivity and a trait marker encompassing increased emotional reactivity, greater depth of processing, and subtle awareness of environment stimuli. Experiences resulting from hypersensation can be distressful and contribute to the occurrence of nightmares, according to the Continuity Hypothesis in which negative experiences in waking life transfer to dreaming, alongside intense negative emotions before sleep [60]. In addition, according to the nightmare theory of the selective mood regulatory function of dreaming [61], the occurrence of nightmares are due to the emotional surge in REM sleep that dreaming fails to adaptively regulate. Based on the findings demonstrating the disruption of REM sleep in ASD [62], it might be the case that REM disruption contributes to the failure of dreaming to adapt waking emotions, which thereby leads to nightmares.

### 4.3. CSHQ/Nightmare Frequency

Although nightmare frequency positively correlated with CBCL, BRIEF, and SCAS in the TD and FASD groups, nightmare frequency was not associated with sleep related measurements from the CSHQ. These measure bedtime resistance, sleep onset delay, sleep anxiety, night waking, parasomnia, sleep disordered breathing, and daytime sleepiness. This may be because CSHQ scores did not contribute to nightmare frequency as sleep quality is generally not a leading factor of nightmare frequency [22]. However, within some subscales of the CSHQ, particularly sleep anxiety, night waking, and parasomnias, caregivers were asked whether during pre-sleep children experienced negative affect, troubling thoughts, or whether children woke up at night screaming or were inconsolable. It would have followed, then, for these items within the CSHQ subscales to correlate with nightmare frequency. Perhaps due to the small number of participants who reported nightmares such data were not found, or this may be due to the ceiling effect of high nightmare frequency and high parasomnia scores. Within previous analyses of the larger dataset [43,44], parasomnias were significantly associated with executive functioning and behavioural outcomes in the TD and FASD group, but less so in the ASD group. Night wakings however were associated with somatic complaints and attention in the TD group, which is comparable to the present analysis. However, this result warrants further investigation.

### 4.4. CBCL/Nightmare Frequency

In the TD group, nightmare frequency was significantly correlated with several CBCL subscores, particularly those related to affect. These were the subscales of anxious/depressed, attention, and aggression. In the FASD group, nightmare frequency was significantly correlated with withdrawn, somatic, anxious/depressed, and thought problems. Influential factors of negative emotions before sleep [18], the susceptibility of dreaming to waking intense emotions [23], as well as the theoretical models of continuity hypothesis and pre-sleep states as the behavioural problems displayed by children could explain the relationship between the manifestation of intense emotional experiences and nightmare frequency and content.

The CBCL assesses children’s withdrawn, somatic, anxious, social, thought, attention, delinquency, and aggressive behaviour, however the instrument itself is used differently between the two clinical groups. Due to the widespread neural damage incurred by prenatal alcohol exposure, children with FASD may have difficulties with a number of behaviours, which are compounded by the environmental stressors common to the psychosocial environment [38]. Children with FASD therefore tend to score within clinical levels in a pattern-like manner on the CBCL, due to developmental, regulatory, affect-based and executive functioning issues, and as a result the CBCL is used as a screening tool for FASD [38]. Behavioural manifestations in ASD are not as homogenous, and children with ASD present with wide range of internalised and externalised behaviours across the several subsections of the CBCL [63]. This heterogeneity may explain why CBCL scores were not associated with nightmare frequency in the ASD group. Within the TD group, the present results are comparable to previous data on nightmare frequency and aggression in children. In the previously mentioned large scale study [26], nightmare frequency was significantly correlated with aggression, as well as mood disturbance and hyperactivity.

### 4.5. SCAS/Nightmare Frequency

TD children were more likely to experience nightmares if they also scored within the clinical range of ‘anxious/depressed’ on the CBCL. In the FASD group, SCAS total score and the subscale of panic symptoms were significantly correlated with nightmare frequency. Anxiety subscales measured panic symptoms, separation anxiety, physical injury fears, social anxiety, obsessive compulsive symptomology, and generalised anxiety. It is reasonable to assume a relationship between anxiety and nightmare frequency given the involvement of pre-sleep emotions on the occurrence of nightmares, as well as the role of emotional continuity and threat simulation [18,23,58]. Anxiety is consistently associated with sleep problems both in children with ASD [28] and FASD [44]. In addition, nightmares are conceptualised as an anxiety-based phenomenon [60]. Additionally, nightmares are connected to emotional regulation [61], fear [64], repressed fear [65], and the transformation of shame into fear [66]. As mentioned above, threatening experiences during wake may be rehearsed during sleep which may explain why anxiety was significantly associated with nightmare frequency within our sample.

### 4.6. BRIEF/Nightmare Frequency

Executive functioning related measurements that were measured related to working memory, emotional control, inhibition, monitoring, organisation of materials, planning and organising, and shifting. Simor et al. [67], for example, refer to the association between executive functioning and nightmare frequency due to impaired fronto-limbic functioning during REM sleep. It is interesting that this association was found only in the TD group and not the clinical groups, both of which are clinical conditions related to frontal lobe functioning, but this is likely due to a higher proportion of the clinical group already reaching maximum executive functioning scores and creating a ceiling effect which was not seen in the TD group.

## 5. Strengths and Limitations

This is the first study to report nightmare frequency and content in children with FASD. Sleep problems in ASD and FASD are currently understudied, particularly those pertaining to behavioural and affect based outcomes. Through further research such as this it may be possible to develop sleep interventions for children with neurodevelopmental conditions. This study additionally benefited from a large sample size from which nightmare data could be extracted, but further studies on nightmares in these clinical populations should take into consideration the smaller subsamples of children who experience nightmares. Some of the strengths of this study are within the large sample size, its novelty, and access to a relatively understudied population of children with FASD.

Several limitations are also of importance here. Firstly, parental reports are subjective views of children’s sleep problems, and further analysis can benefit from objective sleep measures such as actigraphy or polysomnography (PSG). Such objective investigations may be used to correlate PSG or actigraphy data with nightmare frequency or content. Secondly, the results reported here may be attributed to a selection bias, in that parents of children with sleep problems were more likely to want to take part, and the sample may not have had the heterogeneity of a population-based sample. Around a third of participants had to be excluded because of the lack of a formal diagnosis of FASD or ASD, due to a general lack or delay in diagnostic services available in the UK. Thirdly, predictions within this study are statistical descriptions. Without an experimental design and with the limits of cross-sectional data, causation cannot be implied. However, this study has provided a platform for the need for further examination using objective sleep and psychometric measures in these clinical populations.

## 6. Summary

We report that, analogous with theory, nightmares may be associated with waking emotions in children with ASD or FASD, although the causative, bidirectional associations are less clear. The cross-sectional nature of the present data means that any inference about direction of the associations between nightmares and psychometric outcomes can only be hypothetical, and further analysis could possibly assess nightmare content and frequency in greater depth. We recommend the development of sleep intervention strategies to reduce general anxiety, as well as affect, aggression, hyperactive, and other maladaptive behaviours of the child during pre-sleep activities, thereby reducing the occurrence of nightmares.

## Figures and Tables

**Table 1:** Participant details.

	ASD	FASD	TD
***n***	61	112	104
**Male/Female**	46/15	62/50	55/48
**Age (M(SD))**	10.03(2.54)	9.54(2.86)	9.43(2.55)
**Age Range**	6.07–15.56	6.09–15.98	6.47–15.90
**SES ½/3**	17/35/9	14/74/26	23/67/15

**Table 2:** Nightmare content reported by caregivers.

	ASD		FASD		TD	
	n = 67		n = 91		n = 103	
**Typical nightmare themes**						
* Being chased*	2/27	7%	10/67	15%	1/22	5%
* Injured/dying*	3/27	11%	4/67	6%	2/22	9%
* Sensing scary*	1/27	4%	1/67	1%	0/22	0%

**Table 3:** Dream characters in typical nightmare themes.

	Being Chased	Injured/Dying	Sensing Scary
ASD	Aliens	Wolves, Aliens, family members, themselves	
FASD	Wolves, monsters, strangers, someone, large insects	People, mum, toys, dogs	Witches, ghosts
TD	Dog	People, puppy	

**Table 4:** Thematic analysis of nightmare content.

	Separation	Fantasy	Persecuted/Being Chased	Real Life	Death/Injury/Violence	Robbery/Theft/Crime
TD (n)	3	3	2	3	2	0
TD (%)	23%	23%	15%	23%	15%	0%
FASD (n)	11	17	10	5	11	5
FASD (%)	24%	30%	22%	9%	24%	9%
ASD (n)	4	4	2	4	4	0
ASD (%)	22%	22%	11%	22%	22%	0%

**Table 5:** Group differences on CSHQ, SCAS, CBCL, BRIEF.

	ASD	FASD	TD		TD vs. ASD	TD vs. FASD	ASD vs. FASD
	Mean (*SD*)	Mean (*SD*)	Mean (*SD*)	*F*	Sig.		
**CSHQ**							
Total	55.59 (9.22)	56.01 (8.53)	48.27 (8.65)	6.842 **	**0.012**	**0.001**	0.995
Bedtime resistance	9.63 (3.10)	9.19 (2.74)	9.27 (2.66)	0.233	0.960	0.999	0.865
Sleep onset delay	4.74 (2.46)	5.15 (2.73)	3.86 (2.08)	2.108	0.548	0.104	0.853
Sleep duration	7.74 (1.35)	7.43 (1.52)	7.73 (1.12)	0.645	1.000	0.762	0.697
Sleep anxiety	8.93 (2.77)	8.91 (3.24)	5.73 (2.33)	10.157 ***	**0.001**	**0.001**	1.000
Night wakings	7.74 (3.27)	7.64 (2.80)	4.95 (2.44)	8.090 **	**0.003**	**0.001**	0.998
Parasomnias	7.74 (3.19)	8.01 (3.29)	8.05 (4.10)	0.070	0.986	1.000	0.978
Sleep disordered breathing	2.78 (1.31)	2.79 (1.11)	2.50 (1.37)	0.504	0.808	0.673	1.000
Daytime sleepiness	7.70 (3.21)	7.85 (3.77)	7.68 (4.19)	0.025	1.000	0.997	0.997
**SCAS**							
Total	43.04 (16.15)	43.58 (17.34)	29.36 (17.93)	5.977 **	**0.019**	**0.002**	0.999
Panic Attack	6.11 (5.57)	6.45 (4.30)	2.73 (3.49)	5.841 **	**0.029**	**0.002**	0.982
Separation Anxiety	9.30 (3.71)	9.75 (3.98)	6.50 (4.10)	5.693 **	**0.044**	**0.002**	0.940
Physical Injury	6.04 (3.20)	6.24 (3.02)	4.59 (2.72)	2.529	0.262	0.067	0.987
Social Phobia	7.33 (3.08)	8.60 (4.63)	7.05 (4.59)	1.504	0.994	0.344	0.467
Obsessive Compulsive	5.81 (3.73)	4.81 (3.49)	2.86 (3.09)	4.480 *	**0.011**	0.059	0.476
Generalised Anxiety	8.44 (3.83)	7.75 (3.53)	5.64 (3.80)	3.916 *	**0.025**	**0.048**	0.774
T Score	65.37 (4.11)	65.12 (5.06)	57.41 (15.11)	8.759 ***	**0.002**	**0.001**	0.999
**CBCL**							
Total	64.41 (21.88)	77.82 (25.71)	39.27 (26.96)	19.735 ***	**0.002**	**0.000**	0.053
Withdrawn	5.19 (2.90)	6.18 (3.82)	4.00 (4.23)	3.012	0.605	**0.043**	0.543
Somatic Complaints	4.33 (3.79)	4.19 (3.06)	(2.32) ****	7.526 **	**0.004**	**0.001**	0.996
Anxious Depressed	11.89 (6.14)	11.97 (6.55)	7.73 (7.12)	3.680 *	0.085	**0.022**	1.000
Social Problems	6.22 (2.61)	8.12 (2.86)	3.55 (2.94)	22.587 ***	**0.004**	**0.001**	**0.009**
Thought Problems	5.37 (3.15)	5.27 (2.74)	2.55 (2.48)	8.655 ***	**0.002**	**0.001**	0.998
Attention Problems	10.93 (3.74)	12.97 (3.68)	5.82 (4.34)	29.009 ***	**0.001**	**0.001**	0.053
Delinquency Externalising	3.52 (3.17)	6.31 (3.89)	2.50 (2.41)	12.746 ***	0.673	**0.001**	**0.001**
Aggression Externalising	16.96 (7.94)	22.81 (8.51)	11.77 (8.01)	16.136 ***	0.090	**0.001**	**0.006**
**BRIEF**							
Total	175.07 (25.32)	181.43 (21.52)	128.32 (35.14)	36.817 ***	**0.001**	**0.001**	0.599
Working Memory	71.70 (11.37)	74.22 (10.71)	56.57 (12.95)	19.528 ***	**0.001**	**0.001**	0.689
Shifting	76.44 (13.15)	78.59 (12.57)	56.81 (14.88)	22.279 ***	**0.001**	**0.001**	0.848
Planning and Organising	68.41 (10.46)	72.17 (10.18)	55.29 (14.60)	18.038 ***	0.000	0.000	0.355
Organisation of Materials	60.07 (8.46)	61.25 (11.34)	49.00 (12.76)	10.068 ***	0.002	0.000	0.951
Monitoring	67.74 (9.51)	69.70 (9.93)	51.52 (14.22)	23.152 ***	0.000	0.000	0.801
Inhibition	70.44 (11.09)	70.53 (11.09)	55.76 (15.07)	13.058 ***	0.000	0.000	1.000
Initiate	69.44 (9.85)	68.66 (13.25)	52.57 (12.77)	14.734 ***	0.000	0.000	0.989
Emotional Control	71.89 (11.14)	70.17 (13.32)	56.24 (14.41)	10.620 ***	0.000		0.913

* p < 0.05. ** p < 0.01. *** p < 0.001. **** Gabriel post-hoc test further indicated.

**Table 6:** Pearson correlation between nightmare frequency and psychometric outcome.

**TD**									
***CBCL***	*Total*	*Withdrawn*	*Somatic Complaints*	*Anxious Depressed*	*Social Problems*	*Thought Problems*	*Attention* *Problems*	*Delinquency*	*Aggression*
	0.799 **	0.468	0.594 *	0.768 **	0.306	0.668 *	0.807 **	0.344	0.698
***BRIEF***	*Total*	*Working Memory*	*Shifting*	*Planning Organise*	*Organise Materials*	*Monitor*	*Inhibition*	*Initiate*	*Emotional Control*
	0.638 *	0.648 *	0.624 *	0.615 *	0.101	0.575	0.405	0.836 **	0.683 *
**FASD**									
***CBCL***	*Total*	*Withdrawn*	*Somatic Complaints*	*Anxious Depressed*	*Social Problems*	*Thought Problems*	*Attention* *Problems*	*Delinquency*	*Aggression*
	0.444 *	0.502 **	0.358 *	0.405 *	0.318	0.453 **	0.353 *	0.238	0.171
**SCAS**	Total	Panic Attack	Separation Anxiety	Physical Injury	Social Phobia	Obsess/ Compulsive	Generalised Anxiety	T Score	
	0.353	0.413 *	0.138	0.232	0.128	0.289	0.342	0.352 *	

Note. All the bias-corrected and accelerated (BCa) bootstrapped confidence interval did not contain “zero”. * p < 0.05. ** p < 0.01.

## Data Availability

All data pertaining to this paper can be found in Appendix A.

## Appendix A

**Table A1:** All Nightmare Content.

Group	Does Your Child Have Nightmares? If So, Please Describe the Content and Frequency.
TD	Random content. Could be me leaving could be monsters. Could be about going to school. Usually weekly.
TD	Very rarely, last time she dreamed our puppy had died.
TD	At least once a week, they tend to involve wolves, aliens or family members going away/being hurt.
TD	Rarely at the minute and usually he will get up out of bed but not fully awake, needs consoling but will go back to bed easily and fall back to sleep.
TD	May call out for Mummy or Daddy once every couple of weeks. Will sometimes talk in sleep but make no sense.
TD	Occasionally about 1 a month. She can’t remember the content.
TD	Yes, he is just been able to tell us that he dreams that monsters are eating people. More often he wakes crying repeatedly (up to 10 times a night) calling for me and tells us that he feels sad.
TD	Nothing I would call a nightmare, but she calls them nightmares. They sound like random dreams, with some running away.
TD	Separation monthly.
TD	Dead people, ghosts, it can happen a couple of times per month.
TD	Every now and then. Normally about something happening to close family or the pets.
TD	The content can vary, and he doesn’t always remember. Depending on our routine they are infrequent as he suffers from night terrors, so we monitor things closely.
TD	Very often most nights speak about being chased by a dog.
TD	School worries.
TD	Sporadic. Maybe every 3 -5 weeks. No regular pattern. Sometimes link to tv.
TD	Once a year maybe. Sometimes involving monsters.
TD	He has nightmares very sporadically, but he doesn’t want to talk about them.
TD	Occasional due to anxiety, issues at school and stress of preparing for GCSE’s.
FASD	Crying screaming thrashing about not often.
FASD	Sometimes, alone, Vikings.
FASD	He sometimes has nightmares. Maybe once a week.
FASD	Yes. Every night. She wakes up screaming and sweating at least once. She seems phased out and will not talk she will however follow instructions. She can’t remember what her nightmares are about and will not talk about them the next day.
FASD	Few times a month for a few consecutive nights then none for a while. Content is not clear.
FASD	Occasional nightmares, something that may be worrying him.
FASD	Occasionally.
FASD	Yes. Always monsters chasing him. Usually once or twice a week.
FASD	Yes, related to past experiences, about 2–4 times per week.
FASD	He tells me he has nightmares most nights. He dreams people die or he dies. He dreams he is alone and can’t find anyone. He feels he can’t come and get me or call out for help as he thinks there are people in the house who will come and kill him.
FASD	Occasionally about monsters and zombies.
FASD	Regularly. Scary witches. Ghosts.
FASD	One every 2 or so weeks. Dreams of being taken by a stranger.
FASD	Once twice a month, she can never remember but she’s shouting at somebody in her sleep.
FASD	Being chased, maybe twice a week.
FASD	Sometimes, being chased or chasing, fighting others, knives or clowns. Less frequent in last year and a half.
FASD	She will shout out in her sleep and talks a lot.
FASD	Usually involving boys fighting over her, once a week.
FASD	Has nocturnal epilepsy. Used to have when younger. Now has waking fear palpitation.
FASD	Yes, more than 2 to 3 times a week.
FASD	None that he talks about.
FASD	Yes-vivid dreams about zombies and monsters are common.
FASD	She can’t tell me the content. 5 times a week approx.
FASD	Maybe, but she cannot tell us.
FASD	House break in.
FASD	Not sure, will sometimes wake up very upset.
FASD	Being ‘robbed’ from me.
FASD	Not often. Once a month perhaps. Often dreams foxes have eaten our chickens. Or a fox jumps into his bedroom window. Or fox staring at him through his 2nd floor bedroom window. Sometimes dreams of giants smashing things up in his bedroom. Or dreams of thieves breaking in and stealing his toys and precious cuddly toys. Sometimes monsters or dinosaurs.
FASD	Rarely but if does being chased.
FASD	Yes, but she is unable to explain the content.
FASD	Yes, and wets bed at same time. Content is varied but can be being chased, about once a month it wakes her up.
FASD	Yes, about twice a week he will have bad dream. He is usually shouting ‘get off me’ and pleading. We go and stroke his head and tell he’s at home in bed and is safe.
FASD	Yes 1-week approx. The past.
FASD	Sometimes not as frequent at the moment but talks a lot in his sleep.
FASD	Not as bad recently. Used to wake and be inconsolable, sometimes self-harming.
FASD	Approx. Twice a week. Usually about bad guys or monsters.
FASD	Occasionally no idea what they are about she says bad guys.
FASD	Occasionally....Mum dying.
FASD	Yes, big presence in his room, disturbed by noise.
FASD	Not being able to get something.
FASD	2–3 per month he tells me he has had a nightmare, maybe has them more but doesn’t say. They always seem to be about him, his brother and his mum but he can’t explain anymore. Suffered neglect while living with mother because of drug and alcohol misuse.
FASD	Sometimes but not clear what the nightmare was about as she doesn’t remember, or she can’t describe it.
FASD	Sometimes he will say I had a bad dream last night.
FASD	Monsters people in his head telling him to do bad things. At least once a week.
FASD	They have reduced following two and a half years of psychotherapy, but they are nightmares of red buttons, trolls, robots.
FASD	Yes, less frequently now, but approx. Every 3 weeks or so. He is unable to tell us the content.
FASD	Not that he says, he’s only mentioned a few in the past but can’t remember them
FASD	Yes. Very abstract not very specific.
FASD	Possibly 3–4 times a month Usually someone chasing him or catching him.
FASD	Yes, quite regular, about his siblings, the dog, his toys dying.
FASD	Usually twice a week shouting n muttering in sleep is noted but child will not recall a nightmare. Once a fortnight usually a nightmare about her safety or my safety.
FASD	Yes, he does. Can be very large insects chasing him. Being killed, shot.
FASD	Every night.
FASD	Every night. Mostly about me dying or there being people in the house that will kill him. Lots of his nightmares don’t make sense.
FASD	Most night. Dreams about skeletons or the monster that eats babies that lives at school.
FASD	Most nights. Used to be an insomniac before going on risperidone.
FASD	Every night not recalled.
FASD	Monsters 2 times a week.
FASD	Yes at least once a week often every night. People or monsters chasing him.
AUTISM	Not sure if he has nightmares but he shouts out throughout the night and tosses and turns.
AUTISM	1 per week but has gone through phases of having lots. V scared of dark so often needs lights on or gets the fear when wakes at night. When he wakes after midnight, he rarely seems that sleepy and I’m not sure he’s going to go back to sleep. He seems v alert.
AUTISM	Rarely, cannot recall content.
AUTISM	Yes often. He doesn’t usually want to say but he often dreams of someone in the family or him dying. He goes through phases where he will have nightmares and night terrors every night and other phase where he doesn’t have them for a while. He doesn’t like closing his eyes or want to go to sleep for fear of nightmares and if he has one in the night, he won’t go back to sleep for fear of it happening again.
AUTISM	Yes-almost every night. He doesn’t want to describe it when he wakes. Later he may say it’s about something from TV or event that’s happened. He struggles to switch off the fear and so needs us to sleep with him to get back and stay asleep.
AUTISM	Yes, about once a week, they are always linked to abandonment- being left at a railway station or about waking and no one else being at home.
AUTISM	Yes. And infrequent once a week if that.
AUTISM	Aliens are coming to get him!
AUTISM	She says she has usually if someone has shown her something or she has seen something by accident. Can be 3–4 times a week or none. No pattern. Gets scared by noises.
AUTISM	Approx. Once every 2–3 weeks.
AUTISM	Always. Car crashes. Death. Torture. Being left alone.
AUTISM	Yes, recurring after suffering trauma. Can be up to 4 times a week.
AUTISM	2/3 times a night.
AUTISM	Yes, sometimes, but content almost never disclosed, just ‘scary’.
AUTISM	Patches of nightmares, subject not always disclosed. A couple of times a man who followed son and friend in school and in wood and clearing and had fireballs for hands.
AUTISM	I think he sometimes has nightmares, but he is unable to explain the content of his dreams. This occurs probably 2 or 3 times a week.
AUTISM	Sometimes he does; he rarely remembers but they seem to involve him being chased. He went through a rough patch when someone at school told him about ‘Five Nights at Freddie’s’, he was anxious and had nightmares about that for a while.
AUTISM	Nightmares 2–3 times a week usually of some form of harm happening to family member.
AUTISM	Sometimes-only a few times a month. He does talk in his sleep though and sleep walk more frequently.
AUTISM	Over past few months since starting high school has been happening about 10 times a month.
AUTISM	Yes, probably 2 to 3 times a week. Sometimes night terrors but this is lessening with age.
AUTISM	Apparently most nights but he can’t describe them.
AUTISM	Occasional nightmare about being lost and unable to find people or being chased by wolves.
AUTISM	Wakes crying saying had bad dream a hand full of times a year.
